# Relationship between blood lead level and male reproductive hormones in male lead exposed workers of a battery factory: A cross-sectional study

**Published:** 2013-08

**Authors:** Khosro Sadeghniat haghighi, Omid Aminian, Farzaneh Chavoshi, Leila Sadat Bahaedini, Shahin Soltani, Fatemeh Rahmati Najarkolaei

**Affiliations:** 1 *OccupationalSleep Research Center, Baharloo Hospital, Tehran University of Medical Sciences, Tehran, Iran.*; 2 *Health Research Center, Baqiyatallah University of Medical Sciences, Tehran, Iran.*

**Keywords:** *Human reproductive index*, *Lead poisoning*, *Sex hormone*

## Abstract

**Background:** The reproductive system is one of the organs that are affected by lead. Lead can cause loss of libido and fertility in men, and menstrual disturbances and spontaneous abortion in woman.

**Objective:** The purpose of this cross-sectional study was assessment of dose-response relationship between blood lead level (BLL) and sex hormones levels in lead exposed male workers.

**Materials and Methods:** One hundred and thirteen male workers enrolled. All workers had at least 6 months of lead exposure and no history of diseases or conditions affecting reproductive system. Blood lead level and hormones levels measured with atomic absorption spectrophotometer and radioimmunoassay method, respectively.

**Results:** Average duration of exposure was 15.92±6.95 years. Mean BLL was 41.41µg/dl±16.99. All of the sex hormones values were in normal range. Pearson correlation showed that sex hormones levels had no significant correlation with blood lead level. Also, there was no significant difference in sex hormones levels between workers with BLL <40µg/dl and workers whose BLL was >40 µg/dl.

**Conclusion:** This study showed that BLL cannot serve as a predictor of male sex hormonal changes. However, it is not possible to rule out the effect of lead on the reproductive system after long-term exposure.

## Introduction

Chronic exposure to lead can cause multiple organ dysfunctions (1). Chronic exposure to lead is common in many developing countries including Iran. Although the rate of chronic exposure to lead has decreased due to better safety standards, still there are cases exposed to it. The reproductive system is one of the organs affected by lead. The reproductive toxicity of lead on male lead workers has been studied however the results have been inconsistent. Some studies have shown reduced sperm count and motility, but there are some data showing no effect on reproductive capability (2(. 

The cross-sectional study by Alexander *et al *in Canada is probably the best published survey available evaluating semen quality and serum concentrations of reproductive hormones in workers exposed to lead. This study showed that in blood lead level >40µg/dl, no association was found between exposure to lead and sperm morphology, motility, or reproductive hormones (3). There is controversy over hormonal changes and the mechanism of loss of libido. Some animal and human studies have reported androgen reduction due to lead exposure (4-7). In other studies, no changes were observed in androgen and gonadotropin levels (8, 9). 

Also biological threshold is not clear especially in low and moderate exposure levels and it had very broad range. However most of the studies, did not establish a concrete relationship between lead exposure and hormonal changes and sperm abnormalities. This fact makes it difficult to determine a “no-effect” level and an accurate dose-response characterization (3, 10). For this reasons, we assessed the dose-response relationship between blood lead level (BLL), testosterone and luteinizing hormone (LH) levels and the pathophysiological cause of loss of libido in lead toxicity.

## Materials and methods

Our cross-sectional study was conducted on all of the workers (113 male workers) with at least 6 months of lead exposure at a battery manufacturing plant during winter 2007. The product of this factory was bullion lead. In addition to provision of personal protective equipment, the workers were all educated for implications of lead toxicity. Routine surveillance monitoring has been performed annually since 5 years earlier. Some workers did not participate in this study. All male workers with employment duration of more than 6 months were included in the study. 

Inclusion criteria comprised of workers with no history of Kline-Felter, cryptorchidism, orchitis in the past 3 months, HIV infection, chemotherapy in the past year, radiation, orchiectomy, GnRH deficiency, hyperprolactinemia, hemochromatosis, alcohol and drug use, or heavy exercise in the past week; they were not athletes, did not have any chronic diseases in the past 6 months, and had no history of adrenal or testicular mass. Ethical review board of Tehran University of Medical Sciences approved the project and informed consent was obtained from all workers. 

A questionnaire was designed for demographic data such as age, marriage status, duration of occupational exposure, cigarette consumption (yes/no), prescribed drugs, medical history, and the duration of occupation in the plant. One blood sample was obtained via special lead venoject after disinfection of subject's skin, then it was divided into two parts; one poured into heparinized lead free test tube for measuring blood lead level, another was used for endocrine system function evaluation and other routine tests of periodic monitoring. Each sample was about 10 cc. 

The samples were stored in -4^o^C and kept in dry ice during transportation to the laboratory. Hormone analyses were performed in clinical laboratory with commercial routine tests for the serum concentration of the male sex hormones. Testosterone, free testosterone, follicular staining hormone (FSH) and LH were measured with radioimmunoassay method. Blood lead concentration was determined by flameless atomic absorption spectrophotometer. All samples were analyzed at a clinical laboratory supervised by Tehran University of Medical Sciences. The measurements were conducted for three times and the mean was recorded. This project was financially supported by the Tehran University of Medical Sciences.

**Statistical analysis**

Analysis of the findings was done with SPSS software version 11.5. Correlation between serum parameters of endocrine system function (testosterone, free testosterone, FSH and LH) and BLL were examined with Pearson correlation. Moreover based on current OSHA blood lead level standards (40µg/dl) samples were divided in 2 categories (<40 versus ≥40 µg/dl). 

Independent sample T-test analysis was used to examine correlation between serum parameters of endocrine system function and those two categories of BLL. Also, to estimate the effect of potential confounders, linear multiple regression models were used for each of the serum parameters of endocrine system function tests as dependent variables and BLL, age, cigarette smoking, body mass index (BMI) and duration of occupational exposure to lead as independent factors. 

## Results

There were 113 participants and all of them were male workers. Mean age of workers was 40.88±7.07 years. Most of them (98%) were married. 76% of them were in no smoking group.

**Biochemical analysis**

In our study population, the mean blood lead level was 41.41 μg/dl (SD=16.99) and mean sex hormones levels were in normal range. The results are shown in Table I. Pearson correlation analysis showed that age had a significant reverse correlation with blood lead level (p=0.006). FSH had a statistically significant direct correlation with age (p=0.03), duration of occupational exposure (p=0.02) and BMI (p=0.04). Testosterone had a significant reverse correlation with smoking and BMI, while free testosterone had a significant reverse correlation with age and duration of occupational exposure (Table II). 

We compared mean differences of sex hormones levels in two subgroups with different blood lead levels (<40 µg/dl and ≥40 µg/dl), but no significant differences were noted (Table III). Results of multiple regression analysis for assessment of interrelationship of potential confounders showed that lead did not serve as a predictor for sex hormone changes. Results of LH were not significant. (p=0.12) (Table IV).

**Table I:** Biochemical data

**Variable**	**Number**	**Mean ± SD**
Blood lead level (µg/dl)	113	41.41 ± 16.99
LH (pg/dl)	113	6.33 ± 10.75
FSH (pg/dl)	113	3.7 ± 3.85
Testosterone (pg/dl)	113	3.7 ± 1.02
Free testosterone (pg/dl)	113	16.8 ± 8.26

LH: Luteinizing hormone.

FSH: Follicular stimulating hormone.

**Table II:** Relationship between sex hormones and assessed variables

**Variables**	**Age**	**Work duration**	**Blood lead level (µg/dl)**	**Smoking**	**BMI**
LH (pg/dl)	0.177	0.139	-0.031	-0.07	0.06
FSH (pg/dl)	0.203*	0.212*	-0.098	0.03	0.189*
Testosterone (pg/dl)	-0.044	0.131	0.036	-0.187*	-0.383*
Free testosterone (pg/dl)	-0.254*	-0.213*	0.126	0.021	0.045

LH: Luteinizing hormone.

FSH: Follicular stimulating hormone.

BMI: Body mass index (kg/m^2^).

* P<0.05: significant. (Pearson correlation test)

**Table III:** Comparison of sex hormones levels in two subgroups

**Blood lead level (µg/dl)**	**≥ 40µg/dl (Mean ± SD)**	**<40µg/dl (Mean ± SD)**
Number	59	54
FSH (pg/dl)	6.04 ± 4.8	6.65± 14.79
LH (pg/dl)	3.53 ± 2.35	3.88 ± 5.02
Testosterone (pg/dl)	4.79 ± 1.12	4.83 ±0.9
Free testosterone (pg/dl)	17.52 ±8.09	16.04 ± 8.46

LH: Luteinizing hormone.

FSH: Follicular stimulating hormone (Independent Student *t*-test).

**Table IV:** Results for the interrelationship of potential confounders

**Variable**				**Regression coefficient (β)**	**Standard error of β**	**p-value**
Free testosterone						
		Age		-0.220	0.142	0.12
		Work duration		-9.29	0.144	0.51
		Blood lead		2.97	0.047	0.52
FSH						
			Age	0.12	0.186	0.49
			Work duration	0.216	0.186	0.24
			Blood lead	-3.2	0.060	0.61
			BMI	0.554	0.294	0.06
Testosterone total						
	Age			1.098	0.013	0.9
	Blood lead			1.128	0.006	0.8
	BMI			-0.109	0.027	0.000
	Smoking			-0.27	0.98	0.17

LH: Luteinizing hormone.

FSH: Follicular stimulating hormone.

p<0.05: significant. (Multiple regression).

BMI: Body mass index.

## Discussion

In this study, we assessed the effect of lead on male endocrine system. One hundred and thirteen exposed workers participated in this study in which we assessed the effect of lead on LH, FSH, and free and total testosterone levels. Most animal and human studies have shown the effect of lead on the reproductive system. Some studies have shown decline of testosterone and elevation or reduction of LH and FSH (11-14). Although a variety of studies have been performed, but still there is controversy regarding the involvement of reproductive endocrine axis in lead toxicity (5, 7-9, 15, 16). 

The results of this study were similar to the studies that failed to demonstrate the effect of lead on the endocrine axis of the reproductive system. After controlling for potential confounding effects, we did not find any significant relationship between BLL and hormonal indices. Blood lead level is an indication of acute or recent exposure. In the case of the retired workers or workers that are relieved of work for a long period, blood levels of hormones are related to bone lead level, but this relationship in recently exposed workers is weak. Our study population had recent exposure so their BLL represented recent exposure. 

It seems that rather than BLL, total body burden of lead has a strong correlation with its biological effects in the human body. Telisman *et al* showed that delta-aminolevulinic acid dehydratase (ALAD) had a strong relationship with bone lead level and sexual hormones. Otherwise, they did not find any significant relationships between blood lead level and sexual hormones (16). Rebecca *et al* showed that in rats with less than one week exposure to low dose of lead acetate, the level of GnRH mRNA has risen. Also in rats with more than one week exposure to low dose of lead acetate, the level of GnRH mRNA raised but upon increasing the exposure time, rate of GnRH mRNA rising decreased (18).

So, it seems that in long term exposure to toxic agent, acclimatization may occure in the body. Although some animal studies have shown the relationship between BLL and sexual hormones, the limitations of these studies for extrapolating effects and results on human species should be considered. One of the factors that can explain the lack of relationship between BLL and sexual hormones in this cross sectional study might be the answer to why the mean BLL of all exposed workers was 41µg/dl, which is far beyond the reported threshold of BLL=10 µg/dl for hormonal changes by another study (13). This high concentration level may be the reason for detecting no relationship between BLL and hypophysial hormones. 

## Conclusion

This study showed that BLL did not serve as a predictor of male sex hormonal changes. We detected no relationship between BLL and hormonal indices. However, it is not possible to rule out the effect of lead on the reproductive system after long-term exposure.

## Conflict of interest

The authors declare no conflict of interest.
